# Older Adults Automatically Detect Age of Older Adults’ Photographs: A Visual Mismatch Negativity Study

**DOI:** 10.3389/fnhum.2021.707702

**Published:** 2021-08-20

**Authors:** Petra Csizmadia, Bela Petro, Petia Kojouharova, Zsófia Anna Gaál, Katalin Scheiling, Boglárka Nagy, István Czigler

**Affiliations:** ^1^Institute of Cognitive Neuroscience and Psychology, Research Centre for Natural Sciences, Budapest, Hungary; ^2^Doctoral School of Psychology (Cognitive Science), Budapest University of Technology and Economics, Budapest, Hungary

**Keywords:** oddball, visual mismatch negativity (vMMN), facial stimuli, aging, own-age bias

## Abstract

The human face is one of the most frequently used stimuli in vMMN (visual mismatch negativity) research. Previous studies showed that vMMN is sensitive to facial emotions and gender, but investigations of age-related vMMN differences are relatively rare. The aim of this study was to investigate whether the models’ age in photographs were automatically detected, even if the photographs were not parts of the ongoing task. Furthermore, we investigated age-related differences, and the possibility of different sensitivity to photographs of participants’ own versus different ages. We recorded event-related potentials (ERPs) to faces of young and old models in younger (*N* = 20; 18–30 years) and older groups (*N* = 20; 60–75 years). The faces appeared around the location of the field of a tracking task. In sequences the young or the old faces were either frequent (standards) or infrequent (deviants). According to the results, a regular sequence of models’ age is automatically registered, and faces violating the models’ age elicited the vMMN component. However, in this study vMMN emerged only in the older group to same-age deviants. This finding is explained by the less effective inhibition of irrelevant stimuli in the elderly, and corresponds to own-age bias effect of recognition studies.

## Introduction

The information content of the human face encompasses various important pieces of information such as identity, gender, race, age, and emotional state. This set has utmost importance in interpersonal and social behavior. In this study our aim was to investigate the possibility of automatic registration of age by using the visual mismatch negativity (vMMN) component of the event-related potentials (ERPs) of the brain electric activity.

Visual mismatch negativity emerges to visual events that violate the regularities of a stimulus sequence, even if the eliciting stimuli are unrelated to an ongoing task (for reviews see Kimura et al., 2011; Stefanics et al., 2015). VMMN is usually investigated in the passive oddball paradigm. In this paradigm participants perform a visual (or sometimes auditory) task, while the vMMN-related events are presented outside the task’s context as unattended stimuli. The characteristics of the frequent (standard) events of stimulus sequences may acquire representation, even if the characteristics are simple visual features such as color, orientation, spatial frequency, etc. VMMN also emerges to perceptual categories like symmetry (Kecskés-Kovács et al., 2013b) and orderliness (Durant et al., 2017).

The human face is one of the most frequently used stimuli in vMMN research. VMMN is especially sensitive to facial emotions, i.e., rare (deviant) faces expressing a different emotion from the frequent (standard) faces within the same sequence (e.g., Astikainen and Heitanen, 2009; Li et al., 2012; Stefanics et al., 2012; for a review see Kovarski et al., 2017). In case of gender as another facial feature, Kecskés-Kovács et al. (2013a) recorded vMMN to faces of female models within sequences of male faces, and *vice versa*.

In the present study we investigated the possibility of a similar effect, automatic detection of age by showing photographs of faces of models with different ages. Furthermore, we compared vMMN differences between older and younger participants. Investigations of age-related vMMN differences are relatively rare. Nevertheless, this is an important topic, because vMMN provides direct evidence about the sensitivity of automatic registration of environmental regularities, and the putative change of sensitivity with aging. So far in the context of age differences the majority of vMMN studies applied low-level deviancies, and the results are equivocal. Lorenzo-López et al. (2004) investigated vMMN to horizontally drifting sinusoidal gratings and obtained a long-lasting posterior negativity. Whereas in the older group the negativity was different from zero only at the Oz electrode site, it had a broader distribution in younger participants. Tales et al. (2002) presented single and double bars as standard and deviant stimuli. VMMN in the younger group emerged in the 250–400 ms range, but in the older group they obtained vMMN only in the later part of this range. However, using the same method, Stothart et al. (2013) obtained no age-related differences. Recently, we compared older and younger groups in three studies (Gaál et al., 2017; Sulykos et al., 2017, 2018). In our laboratory Sulykos et al. (2017) investigated vMMN to the offset of parts of continuously presented objects. Age-related vMMN difference emerged in the 180–220 ms range, but there was no vMMN difference in the earlier part of this component. In the Sulykos et al. (2018) study checkerboard stimuli were presented. VMMN appeared in the 100–300 ms range in both age groups, but in the later part of vMNN the amplitude was smaller in the older group. In contrast with the simple stimuli of the above studies, Gaál et al. (2017) investigated category-related vMMN, i.e., letters and pseudo-letters. The stimuli were presented in pairs of subsequent fragments, and the two fragments together constituted the stimuli as wholes. The main variable was the duration between the onset of the fragments, therefore the integration effects on vMMN were investigated in the two age groups. The integration period of the fragments was longer in the older group, showing longer stimulus persistence in the elderly. As this review of previous studies shows, with the exception of the Gaál et al. (2017) study, only low-level features were investigated in the context of age-related differences. One of the aims of the present study is to investigate age-related effects of automatic detection in the case of complex stimuli violating sequential regularities. As far as we know, this is the first vMMN study that investigated the sensitivity of an older and a younger group in the domain of human faces.

As another aim of this study, we investigated vMMNs to deviant photographs showing models of the same age as or a different age than the age of the participants. This issue is related to the phenomenon of own-age bias (OAB). As a considerable body of research shows, people are more efficient in recognizing photographs depicting faces of their own age than faces depicting different ages (for reviews see Rhodes and Anastasi, 2012; Wiese et al., 2013). Theories about the OAB proposed that people have more practice in processing faces of others with age similar to their own. This view emphasizes the importance of the different frequency of encounters for people with different ages (He et al., 2011). As an argument for the importance of encounter frequency, the OAB effect is reduced or even absent in groups with considerable experience with other age-groups (Harrison and Hole, 2009; Wiese et al., 2012). It is possible that in a multidimensional system of perception (Valentine, 1991), as an effect of less frequent experience, other-age faces are farther away from the more discriminative central regions on various dimensions. However, besides the frequency of encounter, motivational and social group relations have also been suggested as underlying mechanisms of OAB. This type of theory was originally proposed for the own-race bias (ORB) in face recognition, an effect stronger than OAB (Mukudi and Hills, 2019). Sporer (2001) supposed that ingroup-outgroup differentiation is an automatic process. The categorization-individualization model (Hugenberg et al., 2010) proposed that in an initial processing stage face processing is categorical, and individualization is a process at a subsequent stage. In the case of faces of a different age, processing is frequently restricted to the first stage. However, across different age groups OAB is not perfectly symmetrical. According to results by Bartlett and Leslie (1986) and Wiese et al. (2008), in groups of older participants no OAB emerged.

As results of some OAB studies show, both stages of the hypothesized processes are automatic. This is because following incidental learning of faces (attractiveness or friendliness rating or age estimation, search for a non-facial target feature), subsequent face recognition is similar to the effect of intentional (attentional) learning (Randall et al., 2012; Neumann et al., 2014). To investigate the possibility of automaticity of OAB-related effects and of age-related sensitivity differences, we compared a younger and an older group of participants in a vMMN paradigm with sequences of young standard – old deviant and old standard – young deviant photographs. We applied the method developed by Stefanics et al. (2012) for emotion-related vMMN. Accordingly, we presented four photographs around a central task field. As a modification of the method, to ensure continuous attentional engagement to the task-field, we introduced a tracking task. What did we expect in the present study? On a general level we expected the automatic perception of the models’ age, that is, the appearance of a negative deviant *minus* standard difference potential (vMMN) over the posterior locations within the 200–400 ms post-stimulus latency range. As a more specific possibility, we expected to find an OAB by registering a vMMN difference between the age groups in the young standard – old deviant and old standard – young deviant conditions. According to the categorization-individualization model of OAB (Hugenberg et al., 2010), only the own-age photographs are processed at the level of individual features. Such age-related difference may lead to increased sensitivity to own-age deviants, and accordingly, a larger deviant *minus* standard ERP difference for photographs of models of the same age as that of the participants.

Age difference of photographs *per se* elicits ERP differences. As an example, in a gender categorization task a larger anterior positivity and a smaller anterior negativity emerged to old faces in a younger group, and in the same group a larger late positivity emerged to old faces in a later latency range (Ebner et al., 2011). Therefore, in the present study we compared the ERPs to stimuli of the same age as deviants and standards (inverse control procedure). Face processing is dependent on the orientation of the photographs. Upside-down presentation of faces decreases the effectiveness of face-specific processing (Yin, 1969; for a review see Rossion, 2009). Low-level visual differences are preserved in upside-down photographs, therefore vMMN differences between original and upside-down presentation argue against the role of age-related low-level feature differences. Accordingly, we did not expect deviant *minus* standard ERP difference for upside-down faces.

In summary, our main goal was to study the possibility of automatic registration of age and to investigate age related sensitivity differences by using the visual mismatch negativity. We compared a younger and an older group of participants in a passive oddball paradigm with sequences of young standard – old deviant and old standard – young deviant photographs. According to results of previous vMMN studies with facial features, we expected the appearance of a negative deviant *minus* standard difference potential (vMMN) over the posterior locations within the 200–400 ms post-stimulus latency range and we expected to find an OAB, a larger deviant *minus* standard ERP difference for photographs of models of the same age as that of the participants.

## Materials and Methods

### Participants

Twenty older (60–75 years) participants were selected from a larger pool of available participants. This selection was independent of the potential difference between the deviant *minus* standard ERPs difference, but they had discernible P1 and N1/N170 exogenous components. In the younger (18–30 years) group seven participants were excluded from a starting sample of 27 participants because they had no discernable exogenous components. This way there were 20 participants in each age group (younger adults: 10 women; mean age: 22.0 years, *SD* = 2.34 years, older adults: 11 women; mean age: 68.45 years, *SD* = 3.62 years). Cognitive functions were measured by four subtests (Similarities, Digit Span, Matrix Reasoning, and Digit Symbol-Coding) of the Hungarian version of WAIS-IV (Rózsa et al., 2010). The aggregated mean points were 43.65 (*SD* = 5.85) in the younger group and 52.45 (*SD* = 8.31) in the older group. All participants were right-handed, had normal or corrected-to-normal vision (measured via a Hungarian version of Snellen card), and were free of any kind of neurological or psychiatric disease. Older adults were paid for participation. Younger adults participated in the experiment for course credit, except two paid participants, who were no longer college students. Written informed consent was obtained from all participants prior to the experimental procedure.

The study was conducted in accordance with the Declaration of Helsinki and approved by the United Ethical Review Committee for Research in Psychology in Hungary (EPKEB).

### Stimuli and Procedure

The stimuli were presented on a 24″ LCD monitor (Asus VS229na, 60-Hz refresh rate) on a gray (44.48 cd/m^2^) background at a viewing distance of 1.44 m. ERP-related stimuli consisted of black and white photographs of 16 young and 16 old male models taken from the database constructed by Minear and Park (2004). Using Adobe Photoshop CS3 Extended 10.0 (Adobe Systems Inc. San Jose, CA, United States) the photographs were converted to grayscale (8 bit) and inserted onto a gray background. Each stimulus screen consisted of images of four different individuals, either four young male faces or four old male faces. The photographs appeared on the upper-left, upper-right, lower-left, and lower-right sides from the center of the screen. The average luminance of the faces was 62 cd/m^2^ (*SE* = 1.2 cd/m^2^). The size of the images was 260 × 360 pixels (2.9° × 4.0°). The center of each image was at a 2.7° horizontal and 2.7° vertical viewing angle from the center of the screen. Stimulus duration was 150 ms, the inter-stimulus intervals were between 366 and 416 ms with a jitter in steps of 16.67 ms.

There were four conditions in the experiment in separate blocks (i.e., inverted and upright faces were presented in separate sequences). Photographs were presented either in the original position or inverted (Position: upright, inverted). Either the photographs of young or old models were deviant stimuli (Photographs: young, old). In the sequences 20% of the stimuli were deviants. The order of presentation of conditions was counterbalanced across participants. There were 400 stimuli (320 standards and 80 deviants) within a condition. The presentation order of the models was random with the restriction that a photograph of the same model was not presented at subsequent stimuli, that is, faces changed trial-by-trial. (The photograph of a model as standard face was repeated 80 times, a deviant one was repeated 20 times within a condition).

**FIGURE 1:**
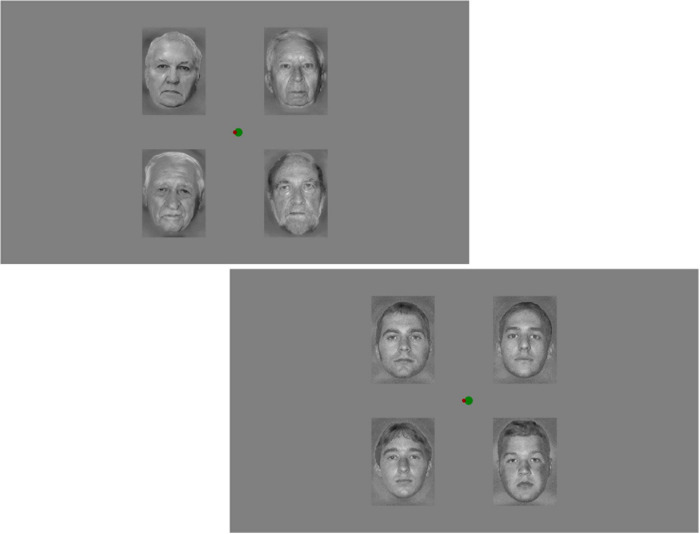
Examples of the stimulus display. At the center there is the task-field with the target and moving circles is at the center.

The task-relevant stimuli appeared on the central area of the screen and consisted of two disks. A red disk served as a fixation point (0.19° visual angle), and a green disk (0.38°) made horizontal pseudorandom movements around the red disk. The participant’s task was to keep the green disk as close to the fixation point as possible using the S (left) and É (right) keys of the keyboard. Errors occurred when the distance of the two disks exceeded 0.77° in either direction. In case of an error, the color of the green disk changed to blue providing online visual feedback. Performance (the sum of the errors in one block) was reported on the screen at the end of each block. Figure 1 shows examples of the stimulus display. The experiment started with a practice block (252 trials) to ensure that the participant fully understood the task. In the practice sequence an equal number of young and old faces were mixed within the sequence. EEG was not recorded in this block.

### Measurement of Brain Electric Activity

Electrophysiological recording was performed in an electrically and acoustically shielded room. Electrical brain activity was recorded from 32 locations according to the extended 10–20 system (BrainVision Recorder 1.21.0303, ActiChamp amplifier, Ag/AgCl active electrodes, EasyCap (Brain Products GmbH), sampling rate: 1000 Hz, DC-70 Hz online filtering). The ground electrode was placed on the forehead (AFz) and the reference electrode was on the nose tip. Both horizontal and vertical electrooculogram signals (HEOG and VEOG) were recorded with bipolar configurations between two electrodes (placed lateral to the outer canthi of the two eyes and above and below the left eye, respectively). The EEG signal was bandpass filtered offline with a non-causal Kaiser-windowed Finite Impulse Response filter (low pass filter parameters: 30 Hz of cutoff frequency, beta of 12.2653, a transition bandwidth of 10 Hz; high pass filter parameters: 0.1 Hz of cut off frequency, a transition bandwidth of 0.2 Hz). Epochs ranging from −100 to 600 ms relative to the onset of stimuli were extracted for all deviants and for those standards that immediately preceded a deviant. The first 100 ms of each epoch served as the baseline. Epochs with larger than 100 μV or smaller than 2 μV voltage change were considered artifacts and rejected from further processing. ERPs were calculated by averaging the extracted epochs (separately for standards and deviants for young and old faces). Difference waveforms were created by subtracting the ERPs to standards from the ERPs to deviants, separately for the two age category of the models (inverse control procedure), i.e., deviant and standard responses to physically identical stimuli were compared (deviant old face vs. standard old face and deviant young face vs. standard young face).

### Analyses and Comparisons

#### Exogenous Components

P1 latency was measured at POz and Oz locations as the largest positivity within the 60–130 ms range, and P1 amplitude was measured as the average of *a* ± 10 ms range around the group averages. Amplitude and latency values were calculated in repeated measure ANOVAs with between group factor of *Group* (younger, older), and within group factors of *Photograph* (young, old), *Stimulus* (deviant, standard), and *Position* (upright, inverted). N1/N170 latency was measured at PO7 and PO8 locations as the largest negative/smallest positive value in the 100–200 ms range, and N1/N170 amplitude was measured as the average of *a* ± 10 ms range around the group averages. In the ANOVAs on latencies and amplitudes, *Location* (left, right) was included as an additional factor. P2 latency (as the largest positive value) was measured within the 170–270 and 190–290 ms latency ranges (younger and older group, respectively), and amplitude was measured as the average of a ± 10 ms range around the group averages at P7 and P8 locations. In the ANOVAs the between group factor was *Group* (younger, older), and within group factors were *Photograph* (young, old), *Stimulus* (deviant, standard), *Position* (upright, inverted), and *Location* (left, right). We report here only age-related differences, because other aspects of exogenous activity are beyond the scope of this study^1^.

#### Difference Potentials

To explore the possibility of deviant *minus* standard differences, as the first step we calculated consecutive *t*-tests (difference from zero as null-hypothesis) at PO7, PO3, POz, PO4, PO8, O1, Oz, and O2 locations on the deviant *minus* standard difference potentials at all points within the 200–400 ms latency range, i.e., in the expected range of vMMN. As criteria we considered significant t-values (*p* < 0.05) at least over two adjacent locations and 20 subsequent significant points (20 ms per location). Afterward we investigated the difference potentials in two epochs: in 230–270 and 330–370 ms, respectively, i.e., the middle part of the 200–300 and 300–400 ms latency ranges. These investigations were conducted in a posterior ROI, containing PO7, PO3, POz, PO4, PO8, O1, Oz, and O2 locations. These tests were conducted only if there were significant results in the exploratory analyzes. In the two epochs we calculated Benjamini-Hochberg corrected *t*-tests, comparing the difference potentials to zero. In these calculations the Statistica 13 (TIBCO Software Inc.) was applied. In case of tendencies of deviant *minus* standard differences, we conducted Bayesian statistics (JASP Team, 2018) to control the reliability of null effects (this calculation was not planned *a priori*). We used the default prior option for the *t*-tests, a Cauchy distribution with spread r set to 0.707. All tests were two-tailed^2^.

In case of reliable differences between the ERPs to deviant and standard stimuli, we conducted a source analysis using the sLORETA method. These results, along with the applied calculations are presented in Supplementary Materials.

## Results

### Behavioral Results

The number of errors (the circle outside the target field) was larger in the older group (36.1, *SE* = 11.9) than in the younger group (2.10, *SE* = 0.60), according to the Mann-Whitney test, *p* < 0.001. The task was easier in the younger group, however, as we noted, participants of the older group attempted to concentrate on the task.

### Event-Related Potentials

#### Exogenous Components

**FIGURE 2:**
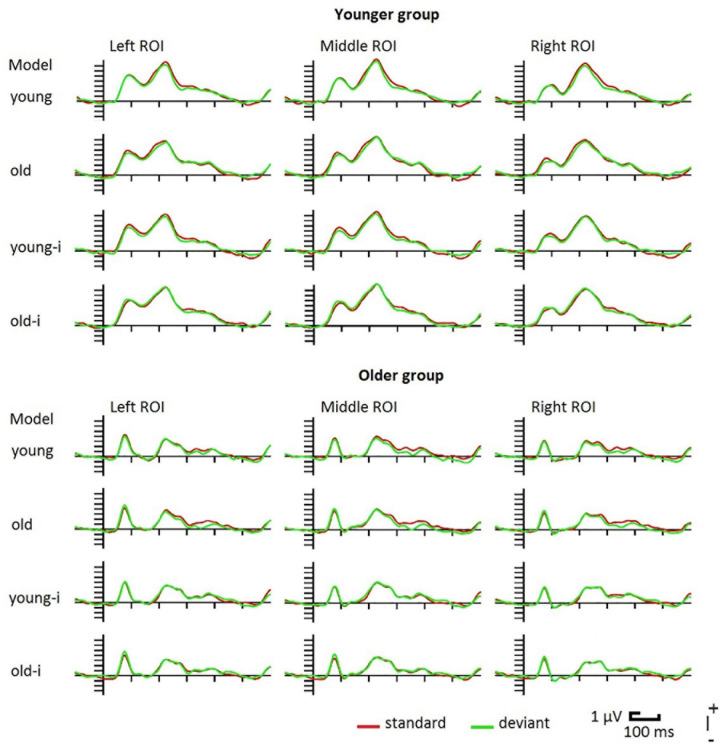
Event-related potentials in the younger and older groups to upright and inverted (-i) photographs of young and old models. For illustrative reasons the posterior ROI is divided into left (PO7, PO3, O1) middle (POz, Oz) and right (PO8, PO4, O2) parts.

As Figure 2 shows, ERPs were different in the two age-groups. Following the P1 component, in the older group the ERP returned to the baseline, and the N1 component was followed by the P2. In the younger group N1 did not reach the baseline. This is because the negativity superimposed on a positive wave, and this positivity peaked as P2. Table 1 shows the latency and amplitude values of these components.

**TABLE 1:** Mean amplitude (μV) and latency (ms) values of the P1, N1, and P2 components for upright and inverted photographs in the younger and older groups to the standard stimuli. P1 was measured at POz, N1 was measured at PO8 and P2 was measured at P7 (S.E.M. in parenthesis).

	**Younger group**				**Older group**			
**Photo**	**Upright**		**Inverted**		**Upright**		**Inverted**	
	**Young**	**Old**	**Young**	**Old**	**Young**	**Old**	**Young**	**Old**
**Latency**								
P1	96 (3.42)	91 (3.43)	91 (3.18)	94 (3.87)	83 (3.00)	79 (2.00)	80 (2.48)	81 (2.90)
N1	139 (4.40)	146 (4.66)	146 (5.34)	149 (4.21)	144 (7.41)	138 (6.84)	139 (6.37)	140 (6.51)
P2	213 (5.06)	207 (4.85)	213 (5.09)	217 (5.12)	247 (6.29)	243 (6.09)	250 (6.17)	244 (6.10)

**Amplitude**								
P1	5.2 (0.74)	4.8 (0.94)	5.1 (0.82)	5.0 (0.91)	3.7 (0.46)	3.7 (0.50)	3.3 (0.43)	3.4 (0.54)
N1	1.4 (0.55)	1.8 (0.60)	1.8 (0.63)	2.1 (0.63)	−0.7 (0.53)	−0.2 (0.90)	−0.4 (0.78)	−0.4 (0.80)
P2	4.2 (0.57)	3.5 (0.66)	3.9 (0.66)	4.0 (0.69)	1.7 (0,55)	2.0 (0.56)	1.7 (0.53)	1.3 (0.62)

On the P1 latency values we obtained a significant main effect of *Group*, *F*(1,38) = 14.66, η_p_^2^ = 0.28, *p* < 0.001, showing shorter P1 latency in the older group. For the P1 amplitude, despite the apparent difference we obtained no age-related difference. For the N1 latency we obtained no age-related differences. It is worth noting that the latencies were below 150 ms, which is shorter than the usual N170 latency. As it is evident from Figure 2, N1 amplitude was larger in the older group, accordingly, this difference was significant, *F*(1,38) = 11.42, η_p_^2^ = 0.23, *p* < 0.01. P2 latency was longer in the older group, *F*(1,38) = 33.60, η_p_^2^ = 0.47, *p* < 0.001, and P2 amplitude was larger in the younger group, *F*(1,38) = 10.15, η_p_^2^ = 0.21, *p* = 0.003.

#### Difference Potentials

In the inverted condition the difference potentials failed to pass the criteria of the exploratory analysis, therefore we did not analyze this condition further. In the younger group the deviant *minus* standard difference just failed the criteria (at the photography with young models, in the 200–300 ms range there were negativities of 28 and 16 ms long epochs at O2 and Oz locations, respectively), therefore we further analyzed the earlier range in this age group. In the older group significant negativity emerged within the 343–374 ms latency range at all locations for the photographs depicting old models.

**FIGURE 3:**
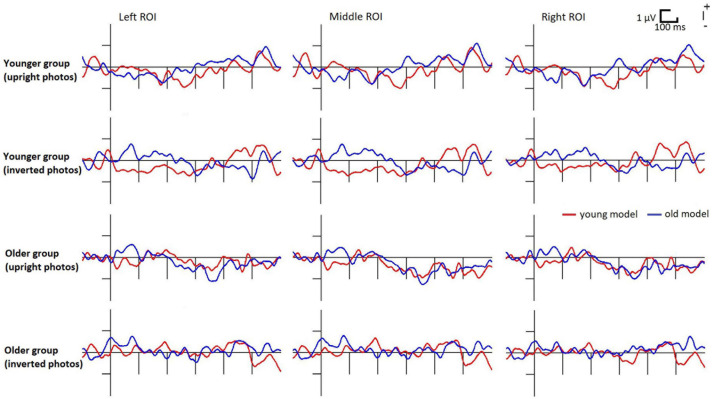
Deviant *minus* standard difference potentials in the younger and older groups to upright photographs of young and old models. For illustrative reasons the posterior ROI is divided into left (PO7, PO3, O1) middle (POz, Oz) and right (PO8, PO4, O2) parts.

**FIGURE 4:**
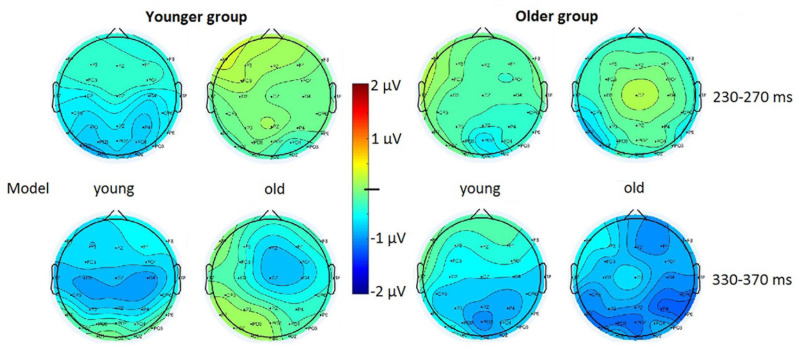
Surface distribution of the deviant *minus* standard difference potentials in the 230–270 and 330–370 ms latency ranges in the younger and older groups to upright photographs of young and old models.

Figure 3 shows the difference potentials, and Figure 4 shows the surface distribution of the difference potentials in the 230–270 and 330–370 ms ranges to upright photographs in the two age-groups for the two ages of models. Table 2 shows the mean amplitude values of the above ranges. In the *t*-tests significant differences appeared in the older group in the 330–370 ms range for the photographs of old models, *t*(19) = 3.76, *d* = 0.79, *p* < 0.05 (Benjamini-Hochberg corrected), and there was a tendency for the negativity for young models *t*(19) = 2.05, *d* = 0.46, *p* < 0.06 (uncorrected). In the younger group we obtained a tendency of young deviant-related negativity in the 230–270 ms range, *t*(19) = 1.85, *d* = 0.41, *p* < 0.08 (uncorrected). No other comparison approached significance.

**TABLE 2:** Mean amplitude of the difference potentials (μV) in the younger and older groups in the 230–270 and 330–370 ms ranges to upright photographs of young and old models (S.E.M. in parenthesis).

	**Younger group**		**Older group**	
	**Young model**	**Old model**	**Young model**	**Old model**
230–270 ms	−0.83 (0.45)	−0.24 (0.29)	−0.36 (0.33)	−0.20 (0.37)
330–370 ms	−0.05 (0.67)	0.09 (0.46)	−0.70 (0.34)	−1.10 (0.31)

In the Bayesian analyses we obtained strong evidence for the negative difference potential in the older group for upright old models in the 330–370 ms range (BF_10_ = 15.93). In this condition an anecdotal evidence appeared in the 330–370 ms range for young models (BF_10_ = 1.31). In the younger group the apparent negativity for the young models in the 230–270 ms range was unreliable (BF_10_ = 0.97).

## Discussion

The aim of the present study was to investigate the possibility of automatic identification of models’ age in photographs. To this end, in a passive oddball sequence of photographs some model’s age were different (deviants) from the frequent age of the models (standards). We investigated a group of younger and a group of older participants with deviant photographs of old and young models, and expected deviant *minus* standard event-related activity, the visual mismatch negativity (vMMN). As a specific expectation, we anticipated different effects to own-age vs. other-age deviancies.

Reliable deviant *minus* standard negativity (using traditional and Bayesian methods) appeared only in the older group to upright photographs of old models. This difference emerged in the 330–370 ms latency range, and it can be identified as vMMN. Although there was a tendency for similar posterior negativity to photographs of young models, the above results are a hint of the own-age effect, i.e., increased sensitivity to infrequent photographs of faces of age similar to that of the participants. Another tendency in the younger group for deviant *minus* standard difference at photographs of young models (in the 230–270 ms range) does not contradict the possibility of larger sensitivity to own-age faces. While the results in the older group corresponded to our expectation, as one of the reviewers noted, another way of thinking leads to different expectation. If participants have higher sensitivity to same-age faces, then it is likely that participants form a more robust standard representation for same-age standards, and as a result, different-age deviants elicit greater vMMN. However, we obtained no results in this direction.

Visual mismatch negativity to face-related stimuli have been reported in various post-stimulus latency ranges. The 320–370 ms range is relatively late, but it is within the range reported in previous studies (e.g., Susac et al., 2004, 2010; Gayle et al., 2012; Kimura, 2012; Vogel et al., 2015) and also within the vMMN range for other complex stimuli like right vs. left hands (Stefanics and Czigler, 2012). Due to the dependence of the position (upright vs. inverted) the effect seems to depend on holistic face processing, instead of the effect of low-level physical differences (e.g., Yin, 1969; Maurer et al., 2002; for review see Rossion, 2009). Our inverse control method, i.e., the comparison of faces of identical age in the role of deviant and standard, underscores this statement.

Using a similar method (four photographs in eccentric positions) Stefanics et al. (2012) obtained much more robust vMMN to emotional deviancy, showing that facial age difference is a less salient characteristic than facial emotion. Being an unexpected result, the sensitivity to deviant photographs in the older group deserves discussion. As a specificity of the present design, four photographs were presented at eccentric locations, and the task in the center of the screen required continuous fixation to the task field. This arrangement required stronger focal attention than other studies in the field of age-related vMMN differences. As a possibility, younger participants concentrated more effectively on the task-field, e.g., they were more efficient in inhibiting the task-irrelevant part of the visual field. On the one hand, this explanation corresponds to the compromised inhibitory processes in some fields of aging research (e.g., Hasher and Zacks, 1988), the larger effect of age-related distraction (e.g., Karthaus et al., 2020), and increased ERP effects of irrelevant stimuli (Kojouharova et al., 2020). On the other hand, spatial attention is relatively preserved in the elderly (for a discussion see Lawrence et al., 2018), and as an example, in the flanker task there is no robust age-related difference (De Bruin and Della Sala, 2018). Furthermore, less effective processing of events appearing at parafoveal regions in older participants is also against the above possibility. As an example, younger participants outperformed older participants in detection of motion direction at parafoveal areas (Park et al., 2020). However, according to some results, irrelevant stimuli outside the focus of attention have larger effects in older adults (Porter et al., 2012; Tsvetanov et al., 2013). As for the vMMN research, in a recent study with younger participants File and Czigler (2019) obtained considerable spatial attention effects on vMMN. In the only study with complex stimuli (meaningful vs. meaningless letter strings; Gaál et al., 2017) the advantage of older participants was due to the longer aftereffect of stimulus appearance. Longer aftereffect may facilitate the more elaborate processing of stimuli. The relatively long vMMN latency supports this assumption. The less efficient filtering of the task-irrelevant stimuli together with the possible advantage in stimulus coding seems to be a favorable condition for our older group for the emergence of vMMN.

As the more specific aim of the present study, the investigation of own-age bias (OAB) in the field of automatic change detection, in the older group we obtained positive results. In this group the magnitude of the reliable vMMN to photographs of old models was similar to the vMMN amplitude in younger groups to emotional face deviants (e.g., Chang et al., 2010; Wang et al., 2014; Sel et al., 2016). As an apparent controversy, in some studies OAB was less pronounced or even absent in older participants (e.g., Harrison and Hole, 2009; Wiese et al., 2012). However, our automatic change-detection procedure is different from the recognition paradigm of OAB studies, apart from a methodological similarity of a certain task that required task-irrelevant coding of facial age (Randall et al., 2012; Neumann et al., 2014). However, even in these studies participants had to attend to other aspects of the faces (e.g., gender, aesthetic value). As results on object-related attention (Duncan, 1984; Scholl, 2001) indicate, even if the ages of the models were task-irrelevant, faces were not “unattended.” On the contrary, in the present study the faces were outside the focus of attention, therefore the faces were not only task-irrelevant, but they were also “unattended.” As the results of the present study show, in the age group with relevant vMMN (i.e., the older group), photographs of their own age were automatically registered as deviant stimuli among the photographs of models of other ages. This way our results show that OAB has a component of automatic sensitivity. On a theoretical (but speculative) level, vMMN is considered as an index of predictive coding mechanism (Stefanics et al., 2014). According to this account, the representation of incoming stimuli is compared to the model of expected events. In case of mismatch, an error signal is compared to gradually updated models throughout a cascade of processes. As Hugenberg et al. (2010) proposed, other-age photographs are processed only at categorical level, whereas for own-age photographs there is an attempt at processing at individual level. The attempt at processing at a deeper level may contribute to a larger discrepancy (surprise) effect and accordingly, to a stronger activity of the match-mismatch mechanism.

Besides the deviant-related ERP differences, we obtained robust age-related differences in the exogenous ERP activities (P1, N1, and P2), i.e., earlier P1 in the older group, and larger and earlier P2 in the younger group. In previous studies the results on age-related differences on P1 are equivocal. Čeponiené et al. (2008) obtained smaller visual P1 in the older group, and this difference was especially large over the occipital regions. In contrast, after controlling for visual acuity (similar to that in the present study), Daffner et al. (2013) obtained larger P1 in older participants. Our results on P1 can be interpreted as preserved early processing in the older group. It is important to remind that the stimuli of the present study were human faces. P1 sensitivity to faces, especially to non-cropped photographs has been reported earlier (e.g., Dering et al., 2011). However, unlike in some studies (e.g., Pesciarelli et al., 2011), in the present study we found no P1 amplitude difference between the upright and inverted faces, showing that in the present study P1 had no strong connection to a face-specific processing stage. Facial stimuli typically elicit the posterior N170 component (e.g., Bentin et al., 1996). The N1 component of the present study was earlier than the usual latency of N170. Furthermore (like in case of P1), we obtained no N1 difference between the upright and inverted faces, e.g., longer N170 latency to inverted faces (Rossion et al., 2000). In an earlier study with similar stimulus presentation (four photographs at the four corners of the visual field) we got characteristic N170 components (Stefanics et al., 2012). As a marked difference between the studies, in the Stefanics et al. (2012) study the target-stimuli appeared intermittently as a change of the fixation cross, whereas the tracking task of the present study required continuous attentive processing. The strict attentional control might diminish the recordable negativity within the 100–200 ms latency range.

In the younger group N1 superimposed on a positivity peaked in the usual P2 range. The function of the processes underlying P2 is unclear, but their role is implicated at different stages of face processing (Itier and Taylor, 2004; Boutsen et al., 2006). Amplitude changes of P2 (P200) appeared in studies that investigated face-related decisions. Wiese et al. (2008) obtained amplitude decrease for old faces at older participants, and they interpreted the difference as deeper or more extensive processing at stimulus ambiguity. Faerber et al. (2015) obtained P2 (P200) amplitude reduction as a priming effect in younger participants, supporting this interpretation. In a passive task, i.e., without intentional decision demand, we obtained no such amplitude difference.

In summary, sequences of photographs showing models of particular age acquire memory representation for this regularity, even if the photographs are irrelevant (unattended). Photographs violating this regularity (deviants) elicit the vMMN component. This process is more effective in older adults, especially for deviant photographs of old models. Exogenous visual components are markedly different in younger and older groups, but little is known about the functional aspects of these differences.

## Data Availability Statement

The original contributions presented in the study are included in the article/Supplementary Material, further inquiries can be directed to the corresponding author/s.

## Ethics Statement

The studies involving human participants were reviewed and approved by United Ethical Review Committee for Research in Psychology (EPKEB). The patients/participants provided their written informed consent to participate in this study.

## Author Contributions

PC, IC, and ZG designed the study. PC, BN, and ZG collected the data. PC, BP, PK, KS, and IC analyzed the data. PC, PK, and IC wrote the manuscript. All authors contributed to the article and approved the submitted version.

## Conflict of Interest

The authors declare that the research was conducted in the absence of any commercial or financial relationships that could be construed as a potential conflict of interest. The reviewer MZ declared a shared affiliation, with no collaboration, with several of the authors (PC and BN) to the handling editor at the time of the review.

## Publisher’s Note

All claims expressed in this article are solely those of the authors and do not necessarily represent those of their affiliated organizations, or those of the publisher, the editors and the reviewers. Any product that may be evaluated in this article, or claim that may be made by its manufacturer, is not guaranteed or endorsed by the publisher.

## Footnotes

1 The complete data set is available in the Supplementary Material.

2 It should be noted, that in a formal sense an ANOVA with factors of *Group* (younger, older), *Photograph* (young, old), *Stimulus* (deviant, standard), and *ROI* (parieto-occipital, occipital) corresponds to the design. However, due to the lack of significant differences in the younger group, it is equivocal to select a proper latency range of measurements. Results of an ANOVA using the range of significant difference between young and old photographs in the older group show only significant Group × Picture interaction: *F*(1,38) = 4.13, *p* < 0.05, η_p_^2^ = 0.10.
