# Supplementary material for: Older Adults Automatically Detect Age of Older Adults’ Photographs: A Visual Mismatch Negativity Study
Source: Front Hum Neurosci. 2021 Aug 20;15:707702. doi: 10.3389/fnhum.2021.707702 (PMC8417827; doi:10.3389/fnhum.2021.707702)
Supplement: Supplementary file 4 [file Data_Sheet_1.docx]

The source signal of the average ERP time series was reconstructed on the cortical surface by applying the sLORETA inverse solution (Pascual-Marqui, 2002). The sLORETA gives a solution for the EEG inverse problem by applying a weighted minimum norm estimation with spatial smoothing and standardization of the current density map. The forward model was generated on a realistic BEM head model (Gramfort et al., 2010) by applying a template MRI (ICBM152; 1 mm3 voxel resolution) with template electrode positions. The reconstructed dipoles (pA/m) were determined for every 15 002 sources in three orthogonal directions (unconstrained solution). For each subject the sources were estimated, their difference (deviant *minus* standard) computed, then normalized to baseline and flattened. The differences were calculated only for intervals of reliable ERP differences and compared to 0 with parametric one-sample χ2 -tests for unconstrained sources. The differences were reported as significant if at least twenty voxels exceeded the Bonferroni corrected alpha level (alpha = 0.05). Brain regions for the corresponding significant activations were identified based on the parcellation scheme introduced by Klein and Tourville (2012). The sLORETA analysis was performed with Brainstorm (Tadel et al., 2011).


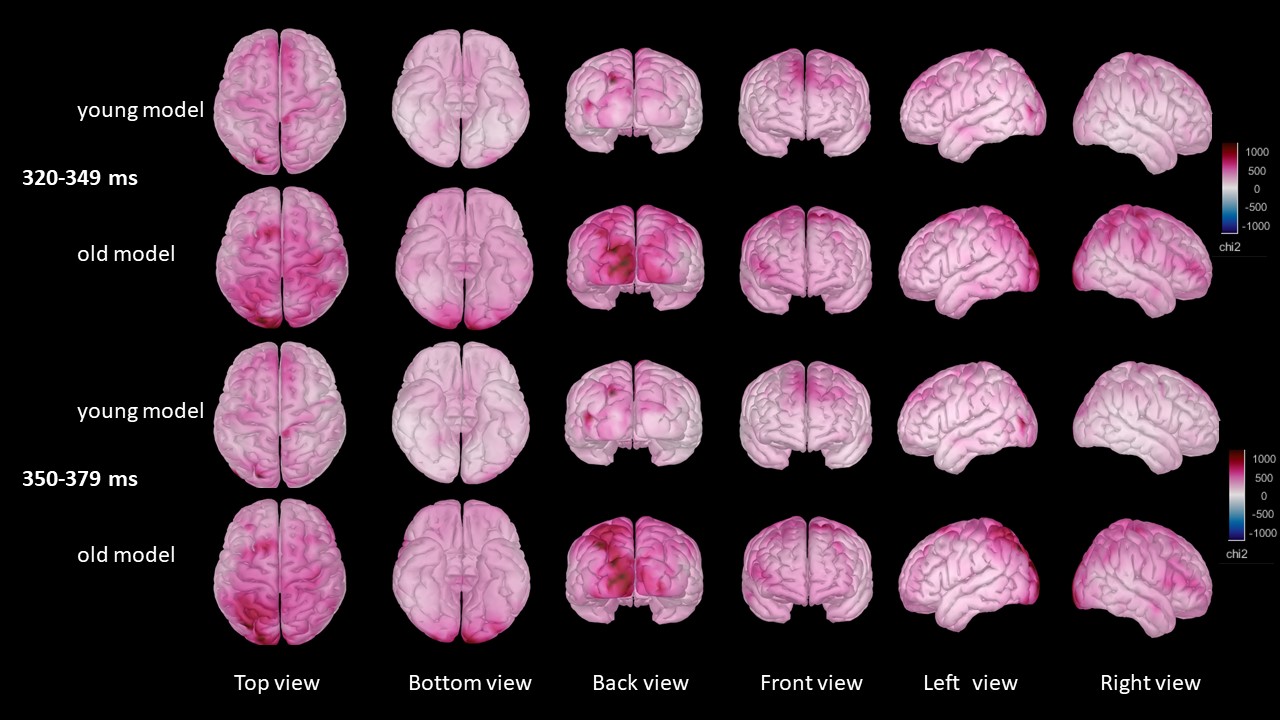


*sLORETA calculation of the difference potentials in the older group for the deviant minus standard activity difference to upright photographs of young and old models (significant differences in the 320-349 ms and 350-389 ms ranges; χ2-test values of the differences). Darker color indicates larger χ2-test values. The differences were calculated only for intervals of reliable ERP differences and compared to 0 with parametric one-sample* *χ2 -tests for unconstrained sources.*

The figure shows the sLORETA localization of the difference potentials in two 30 ms-long epochs within the 320-380 ms range in the older group to photographs of old and young models. Our analysis showed significant differences for the whole cortex. This is one of the possible outcomes as the parametric one-sample χ2-test is a very sensitive test (Tadel et al., 2016). To isolate the areas with greatest difference, for each time interval we determined the voxels for which the χ2-test values were above the mean plus two standard deviations as well as the regions that had at least 20 voxels with such values. As Figure 5. shows, within this range the activity of the older participants was more widely spread for the photographs of old models. For the photographs of young models the activity concentrated on anterior (left middle-frontal, left and right superior frontal, right precentral), parietal (left and right precuneus, left superior and inferior parietal, right postcentral) and posterior (left lateral occipital) structures. For the photographs of old models increased activity to the deviants appeared also in anterior (left superior frontal), parietal (left inferior and right inferior parietal, left and right superior parietal, right postcentral, left cuneus) and posterior (left and right lateral occipital, left pericalcarine) structures (for details see the Excel table in the Supplementary materials).

REFERENCES

Gramfort, A., Papadopoulo, T., Olivi, E., and Clerc, M. (2010). OpenMEEG: opensource software for quasistatic bioelectromagnetics. Biomedical Engineerig, OnLine 9, 45.

Klein, A., and Tourville, J. (2012). 101 Labeled Brain Images and a Consistent Human Cortical Labeling Protocol. Frontiers in Neuroscience. 6, Article number 171.

Pascual-Marqui, R.D. (2002). Standardized low-resolution brain electromagnetic tomography (sLORETA): technical details. Methods and Findings in Experimental and Clinical Pharmacology, 24*.* Supplement D, 5–12.

Tadel, F., Baillet, S., Mosher, J.C., Pantazis, D., and Leahy, R.M. (2011). Brainstorm: A User-Friendly Application for MEG/EEG Analysis. Computational Intelligence and Neuroscience, 2011, Article number 875716.

Tadel, F., Bock, E., Pantazis, D., Mosher, J., Leahy, R., and Baillet, S. (2016). Tutorial
27: Workflows. Available online at: https://neuroimage.usc.edu/brainstorm/
Tutorials/Workflows (accessed April 28, 2021).
